# Virtual Reality Games and the Role of Body Involvement in Enhancing Positive Emotions and Decreasing Anxiety: Within-Subjects Pilot Study

**DOI:** 10.2196/15635

**Published:** 2020-06-17

**Authors:** Federica Pallavicini, Alessandro Pepe

**Affiliations:** 1 Department of Human Sciences for Education University of Milano-Bicocca Milan Italy

**Keywords:** virtual reality, virtual reality gaming, video games, emotions, positive emotions, anxiety, state anxiety

## Abstract

**Background:**

In the last few years, the introduction of immersive technologies, especially virtual reality, into the gaming market has dramatically altered the traditional concept of video games. Given the unique features of virtual reality in terms of interaction and its ability to completely immerse the individual into the game, this technology should increase the propensity for video games to effectively elicit positive emotions and decrease negative emotions and anxiety in the players. However, to date, few studies have investigated the ability of virtual reality games to induce positive emotions, and the possible effect of this new type of video game in diminishing negative emotions and anxiety has not yet been tested. Furthermore, given the critical role of body movement in individuals’ well-being and in emotional responses to video games, it seems critical to investigate how body involvement can be exploited to modulate the psychological benefits of virtual reality games in terms of enhancing players’ positive emotions and decreasing negative emotions and anxiety.

**Objective:**

This within-subjects study aimed to explore the ability of commercial virtual reality games to induce positive emotions and diminish negative emotions and state anxiety of the players, investigating the effects of the level of body involvement requested by the game (ie, high vs low).

**Methods:**

A total of 36 young adults played a low body-involvement (ie, *Fruit Ninja VR*) and a high body-involvement (ie, *Audioshield*) video game in virtual reality. The Visual Analogue Scale (VAS) and the State-Trait Anxiety Inventory, Form-Y1 (STAI-Y1) were used to assess positive and negative emotions and state anxiety.

**Results:**

Results of the generalized linear model (GLM) for repeated-measures multivariate analysis of variance (MANOVA) revealed a statistically significant increase in the intensity of happiness (*P*<.001) and surprise (*P*=.003) and, in parallel, a significant decrease in fear (*P*=.01) and sadness (*P*<.001) reported by the users. Regarding the ability to improve anxiety in the players, the results showed a significant decrease in perceived state anxiety after game play, assessed with both the STAI-Y1 (*P*=.003) and the VAS-anxiety (*P*=.002). Finally, the results of the GLM MANOVA showed a greater efficacy of the high body-involvement game (ie, *Audioshield*) compared to the low body-involvement game (ie, *Fruit Ninja VR*), both for eliciting positive emotions (happiness, *P*<.001; and surprise, *P*=.01) and in reducing negative emotions (fear, *P*=.05; and sadness, *P*=.05) and state anxiety, as measured by the STAI-Y1 (*P*=.05).

**Conclusions:**

The two main principal findings of this study are as follows: (1) virtual reality video games appear to be effective tools to elicit positive emotions and to decrease negative emotions and state anxiety in individuals and (2) the level of body involvement of the virtual video game has an important effect in determining the ability of the game to improve positive emotions and decrease negative emotions and state anxiety of the players.

## Introduction

### Background

As stated by Bowman and Tamborini [1], “Video games can’t be understood divorced from their role as technology that persistently pushed the limits of communication.” While in the 1970s, video games first emerged as simple activities that involved using a keyboard or dial to control tiny black and white objects on the screen, today’s computer games are increasingly and dramatically advanced in terms of both graphics and interaction.

One of the most important points in time in the evolution of contemporary gaming is represented by the entry into the mass market of virtual reality video games in the last few years, following the mass diffusion of commercial head-mounted display (HMD) devices, such as Oculus Rift (Facebook Technologies), Vive (HTC Corporation), and PlayStation VR (Sony Interactive Entertainment) [2]. The popularity of virtual reality video games is continuously growing among users: in 2019, Oculus Quest, the first stand-alone headset (ie, it does not require any external device to work) sold out across multiple stores a week after launching [3]; as well, for the first time, the number of connected virtual reality users on the online gaming platform Steam had surpassed 1 million, as determined by data captured over the course of a month [4].

Compared to more traditional (ie, desktop display device) video games, virtual reality games have profoundly different characteristics [2,5,6]. In particular, one of the main relevant differences is the level of immersion, defined as a “quantifiable description of a technology, which includes the extent to which the computer displays are extensive, surrounding, inclusive, vivid and matching” [7]. Technologies can immerse their users in a virtual environment to different degrees, from a simple nonimmersive presentation on a computer screen (ie, desktop displays) to immersive systems, such as HMDs like Oculus Rift (Facebook Technologies) or Vive (HTC Corporation) [2]. The sense of immersion into mediated computerized environments, in general, and computer games, in particular, has previously been explained through spatial presence and flow [8,9]. Even if these concepts share conceptual similarities, such as immersive components and intense feelings, they refer to different theoretical constructs [8]. In particular, while presence relates to a sense of spatial immersion in a mediated environment [10], flow is generally defined as the optimal experience when nothing else matters [11,12]. With respect to computer games, flow has been defined as the sensation of influencing the activity in the virtual world (ie, *gaming in action*) [13], and it is recognized as a central element of exciting gaming experiences [14,15].

Interestingly, scientific studies have recently started to recognize the potential positive impact of virtual reality video games on people’s health (eg, [16,17]) and cognitive abilities (eg, [18,19]). For example, a previous study [18] has reported the efficacy of a virtual reality exercise-based dance game, *DANCE*, that was created ad hoc by researchers for the training of executive functions in older people. In addition, another recent study [19] showed the feasibility of using a commercial virtual reality game to assess executive functions and cognitive abilities, as measured by a traditional neuropsychological test, the Trial Making Test (TMT) [20]. The results showed that user performance in the dance game *Audioshield* (Dylan Fitterer) predicted the TMT scores (ie, time to complete TMT-A and TMT-B) [19].

Not only are virtual reality video games potentially useful tools for the assessment and training of cognitive abilities, recent studies have also reported that they can be effective tools for people’s emotional well-being. In fact, previous studies have suggested that virtual reality video games induce more emotion than do games with less-immersive technologies, such as those on a desktop display device (eg, [2,6,21-23]). With few exceptions [24,25], a higher intensity of emotional response has been observed in virtual reality games compared to desktop games, at both a psychological and a physiological level [2,6,21-23]. For example, players reported a more intense emotional experience while playing the first-person shooter (FPS) game *Half-Life 2* (Valve Corporation) in an immersive modality through an HMD compared to playing the same game on a monitor [22].

However, less is known about the ability of a virtual reality game to elicit positive emotions and decrease negative emotions and anxiety in the players, because specific studies on that subject have not yet been conducted.

### Virtual Reality Video Games, Positive Emotions, and Relaxation

One of the most commonly reported motives for playing modern video games is the pleasure offered by digital games: people look for and are more willing to buy games that elicit positive emotions [26-28]. Positive emotions are considered to form the basis for the growing and flourishing self [29] and are especially important in increasing the level of subjective well-being [29-31]. As stated by broaden-and-build theory [32-34], “...certain discrete positive emotions—including joy, interest, contentment, pride, and love—although phenomenologically distinct, all share the ability to broaden people’s momentary thought-action repertoires and build their enduring personal resources, ranging from physical and intellectual resources to social and psychological resources” [34].

Several studies have shown that computer games played on desktop display devices can generate positive emotions, such as joy or happiness (eg, [35-37]). For example, in a study investigating event-specific emotional responses while playing *Super Monkey Ball 2* (SEGA)—a platform game (ie, a subgenre of an action game) where the player controls a character who has to jump and climb between suspended platforms while avoiding obstacles—players who experienced not only positive events (eg, acquiring in-game goods) but also some negative events (eg, falling over the edge of the game board) were reported to elicit positively valenced arousal [26]. In another study, predominantly positive emotions were reported while playing the action game *Counter-Strike* (Valve Corporation), as assessed by an electroencephalogram (EEG) alpha index; in addition, subjective ratings of emotional responses indicated happiness, both during and after the game [37]. Furthermore, dopamine, a neurotransmitter linked to sensations of pleasure and reward, has been detected using positron emission tomography (PET) during video game playing [38]. In addition, regular players have advocated video game play as a means of relaxation [39-41], and the search for distraction and escapism is among the most often reported motives for playing video games [42-44].

Different types of desktop video games have been reported to be effective in decreasing anxiety in individuals, inducing a state of relaxation (eg, [45-49]) and positive emotions [50]. For example, video games characterized by low cognitive load and generally short time demands, such as *Tetris* (Nintendo) or *Angry Birds* (Rovio Entertainment Corporation), have proven to be able to diminish state anxiety [51,52] as well stress in players [41], even more effectively than traditional techniques, such as medical treatment [51,52] or guided meditation [53,54].

Interestingly, some recent studies have reported a greater increase in positive emotions after playing a virtual reality game compared to a game on a desktop display [2,23,55]. For instance, players showed a higher level of happiness and surprise, as assessed by self-report questionnaires, after playing an FPS game (ie, *Smash Hit* [Mediocre AB]) in virtual reality versus after playing the same game on a desktop display [2]. In addition, while the perceived feeling of happiness increased after playing the bestselling survival horror game *Resident Evil: BioHazard* (Capcom) in virtual reality, the opposite result was obtained after desktop gameplay, specifically, a decrease in the sense of happiness in comparison to the baseline [55].

Thanks to virtual reality’s unique features of immersion and body involvement [56,57], virtual reality games seem to be appealing new tools to elicit positive emotions and to decrease anxiety in individuals. However, until now, no study has been conducted specifically to test the ability of this new type of interactive video game to enhance positive emotions and to decrease negative emotions and anxiety in individuals.

### Body Involvement and Psychological Benefits of Video Games

In addition to differences in terms of immersion, what strongly distinguishes virtual reality video games from traditional ones is that in virtual reality, the movement of the player’s body itself becomes the main interface for interacting with the virtual world [2,58,59]. In a virtual reality video game, in fact, the player can interact with virtual content not only through a joypad or a keyboard but also by using head rotation, eye movements, or specially designed controllers that respond to the position and movements of the player in a defined space [2]. Depending on the specific video game and virtual reality system adopted, the player's level of body involvement can vary significantly. For example, in *Smash Hit VR* (Mediocre AB), which is compatible with the HMD Gear VR (Samsung), it is possible to interact in the game using only head movements. In contrast, in other titles, such as *Beat Saber* (Beat Games) or *Superhot VR* (SUPERHOT Team), the movement of the whole body is required to play the game.

Studies from various disciplines have investigated the relationship between body movement and well-being, showing that body movement affects emotional processes, with an almost immediate antianxiety and antidepressive effect [60-64] and with long-term positive outcomes on physical health [65-68].

Interestingly, previous studies have reported that an increased involvement of the body while playing video games on desktop displays leads to more intense emotional and affective responses by the player [69,70]. Furthermore, recent preliminary studies have reported that exergames—ones that are considered a combination of video gaming and physical exercise requiring physical effort from the player in order to play the game [71-73]—are able to elicit positive emotions among older adults [71], inducing higher positive emotions than traditional exercise [74], and are able to reduce state anxiety in a nonclinical sample of healthy women [75].

These considerations about the critical role of body movement in individuals’ well-being and in emotional responses to video games make it critical to understand how body involvement can be exploited to modulate the psychological benefits of virtual reality games in terms of enhancing players’ positive emotions and decreasing their negative emotions and anxiety.

### Study Objectives

Within the context described above, this within-subjects study aimed to explore the ability of commercial virtual reality games to induce positive emotions and diminish state anxiety of the players, investigating the effects of the level of body involvement requested by the game (ie, high vs low).

The main hypotheses of this study were as follows:

1. Hypothesis 1: Video games played in virtual reality will increase self-reported positive emotions (ie, joy and happiness) and will reduce negative emotions (ie, fear and sadness) and perceived state anxiety.
2. Hypothesis 2: A game with high body involvement (ie, *Audioshield* [Dylan Fitterer]) will elicit stronger positive emotions and a more intense decrease in negative emotions and state anxiety compared to a game with low body involvement (ie, *Fruit Ninja VR* [Halfbrick Studios]).

## Methods

### Participants

The execution of the experiment occurred between July and October 2018. No credits or economic rewards were provided during the research. Participants were recruited from among the students and personnel of the University of Milano-Bicocca and other universities in Milan, Italy, via flyers distributed to campuses and word of mouth. In order to be included in the study, individuals had to meet the following criteria: (1) be aged between 18 and 35 years, (2) have no significant visual impairment (ie, all with normal or corrected-to-normal visual acuity), and (3) have no previous experience with the video games selected for the study (ie, *Audioshield* or *Fruit Ninja VR*). With regard to the size of the sample, a power sample analysis was conducted with a level of statistical significance equal to 5%, power set at 80%, and a medium-sized effect (ie, 0.50) expected at the upper bound [76]. An effect size equal to 0.50 corresponded to a 66% probability that persons from a given condition would experience a higher effect than persons from the other condition, if both were chosen at random. In addition, the effect size value of 0.50 was in line with other similar studies in the field of virtual reality in relation to emotional domains that highlighted a magnitude of effect size that can be considered medium to large. As a result, the suggested sample size for this kind of analysis and research design was set to 34. Before participating, all participants were provided with written information about the study and were required to give written informed consent in order to be included. The study received ethical approval from the Ethical Committee of the University of Milano-Bicocca. The research was conducted in accordance with the American Psychological Association’s 2010 ethical principles and code of conduct. A total of 39 individuals were assessed for eligibility to participate in the study: 3 were excluded (8%) due to not meeting inclusion criteria and no subjects declined to participate. The final sample included 36 participants: 13 females (36%) and 23 males (64%); mean age of 25.6 years (SD 4.18); and mean length of education of 15.9 years (SD 3.1).

### Psychometric Assessment

At the beginning of the experimental session, a self-report questionnaire was given to participants that included the following items:

1. Demographics: participants were asked to indicate their gender (female or male), their age, and their years of education.
2. Gaming habits and previous virtual reality experience: individuals were asked to report on their gaming habits (ie, mean hours spent gaming per week) and to assess their previous experience with virtual reality systems on a 7-point Likert scale ranging from 1 (not at all) to 7 (very much).

In addition, to assess the self-reported indexes concerning positive and negative emotions as well as state anxiety, the following questionnaires were used:

1. Visual Analogue Scale (VAS) [77]: this scale consists of a horizontal line, 10 cm in length, anchored by word descriptors at each end. Participants marked the point on the line—from 0 to 100—that they felt visually represented their perception of their current level of happiness (VAS-HP), surprise (VAS-SP), fear (VAS-FE), sadness (VAS-SD), and anxiety (VAS-A). A large number of studies have confirmed the reliability and validity of VAS measurements (eg, [78-81]).
2. State-Trait Anxiety Inventory, Form-Y1 (STAI-Y1) [82-84]: individuals were asked to specify to what extent, on a 4-point Likert scale ranging from 1 (not at all) to 4 (very much), they perceived at that moment each of the 20 indicated feelings. The STAI-Y1 scale has high internal consistency, with a Cronbach α coefficient ranging from .86 to .95 [83,85,86]; this is considered a reliable measure to capture rapid state-dependent variations in anxiety [85,86].

### Video Games

The video games tested in the study were as follows:

1. *Audioshield* (Dylan Fitterer): this is a virtual reality exergame launched in April 2016 for Vive (HTC Corporation). This title is a dance game in which orbs come flying toward the player, who needs to follow the beat of the music to successfully hit them. The player uses Vive's handheld motion-sensing controls to operate two shields, blue and red: red balls must be deflected with the red shield controlled by the right hand, while blue balls must be deflected with the left hand; purple orbs require a combination of both arms. During the game, the color and the direction of the orbs that the player has to hit changes continuously; for example, the blue balls can come from the right, or red from the left, and require the user to respond correctly very quickly.
2. *Fruit Ninja VR* (Halfbrick Studios): this game represents the 2016 virtual reality version of the bestselling video game *Fruit Ninja* (Halfbrick Studios), a 2010 game for mobile devices where players were required to hit fruit by dragging their finger on the screen. In *Fruit Ninja VR*—a game compatible with Vive (HTC Corporation), Oculus Rift (Facebook Technologies), and PlayStation VR (Sony Interactive Entertainment)—the players used the handheld motion-sensing controls as swords to slice fruits, sitting or standing, and simulating slashes as if they were performing them in real life. The goal was the same as in the mobile version: cut the fruit, do not let it fall, and avoid the bombs in order to collect as many points as possible and climb the rankings.

These video games were selected since, despite the common aim of both games to hit objects that appear in front of the player, they differ profoundly in terms of the body movement required by the game. In particular, while *Audioshield* requires the player to move the whole body in the game space (eg, lateral movements to avoid obstacles and arm movements to hit the balls), in *Fruit Ninja VR* the interaction takes place only through the movements of the superior limbs. In this title, in fact, the position of the feet and superior limbs are fixed at a certain point in space, indicated graphically within the game.

Regarding the hardware, both video games were played on the same virtual reality setting, which included the following units (see Multimedia Appendix 1):

1. Vive system (HTC Corporation): a consumer-grade virtual reality system designed for use in video games. This system consists of an HMD, two controllers, and two infrared laser-emitter units. The headset offers a nominal field of view of about 110 degrees (approximately 90 per eye) through two 1080×1200-pixel displays that are updated at 90 Hz. Games played with the Vive system allow physical movement within a play area that is limited to 4×4 meters.
2. A portable computer: MSI (Micro-Star International) GT73VR, Intel Core i7 processor, GeForce GTX 1070 8GB, 17.3-inch full HD (high-definition) 1920×1080-pixel display.

### Experimental Design

A within-subjects design was used to compare the emotional response, in terms of self-reported positive and negative emotions, and the perceived sense of anxiety among participants in the two experimental conditions. Specifically, the study compared the following:

1. High body-involvement condition (*Audioshield*): after a brief explanation of the video game by the experimenter, the participants were asked to play for about 5 minutes—2 minutes of practice using the song Engage (difficulty: normal; shield: gladiator; environment: horizon), followed by 3 minutes of play to complete the song I Drop Gems (difficulty: normal; shield: gladiator; environment: horizon).
2. Low body-involvement condition (*Fruit Ninja VR*): the participants were asked to play for about 5 minutes—2 minutes of practice using the Zen mode of the video game; individuals were then asked to play twice in the classic mode, where they had to cut as much fruit as possible and prevent it from falling to the ground and losing a life: players spent a mean of 78 seconds (SD 18) for each play.

### Procedure

After individuals gave written informed consent to participate, they completed the self-report questionnaire assessing demographics, gaming habits, and virtual reality knowledge, as well as the VAS-HP, VAS-SP, VAS-FE, VAS-SD, VAS-A, and STAI-Y1. Once this phase was completed, the Vive system was connected to the PC through a 5-meter cable with an HDMI (high-definition multimedia interface) connection and a USB 2.0 connection, and the system was connected to a power source. Participants were asked to wear the HMD and were given the Vive system controllers.

**Figure 1:**
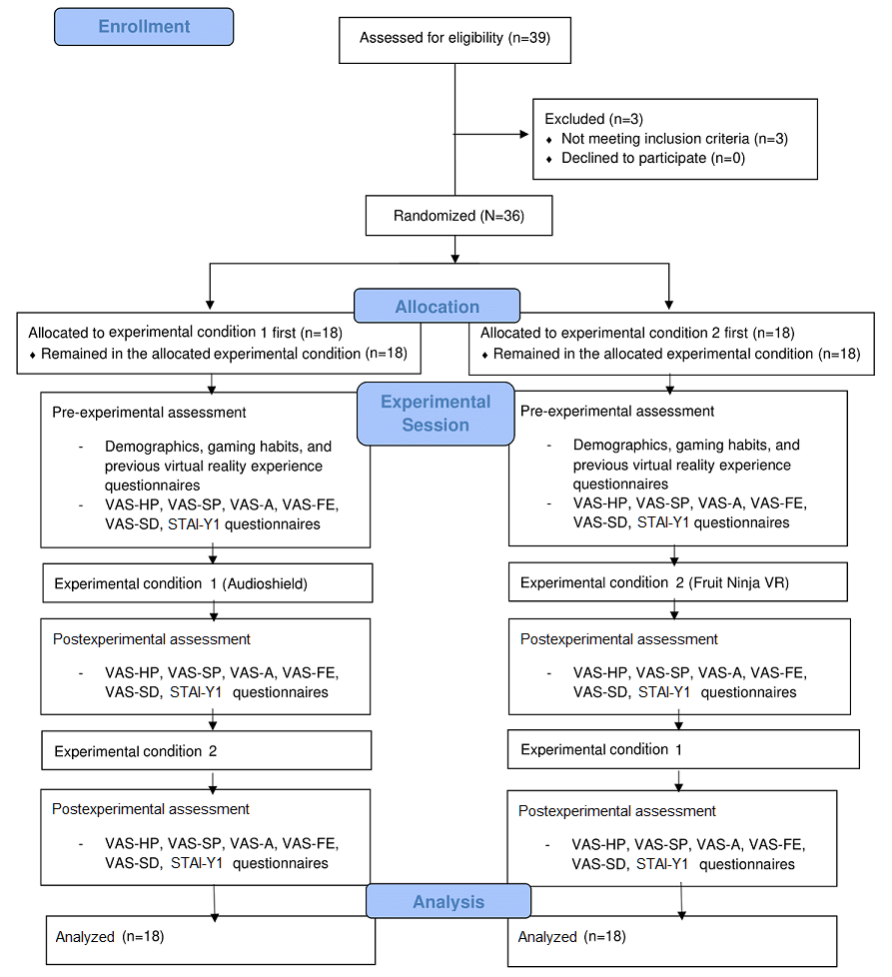
Flowchart of the study procedure. STAI-Y1: State-Trait Anxiety Inventory, Form-Y1; VAS-A: Visual Analogue Scale-anxiety; VAS-FE: Visual Analogue Scale-fear; VAS-HP: Visual Analogue Scale-happiness; VAS-SD: Visual Analogue Scale-sadness; and VAS-SP: Visual Analogue Scale-surprise.

The audio level was set to 45 for all participants. Subjectively, tracking appeared stable when using this configuration, and the video game was playable with no visible tracker artifacts. All measurements were taken in an 8×5-meter room with a 3.2-meter-high ceiling lighted by fluorescent lighting with no reflective surfaces and no exposure to natural light. In the center of this room, a 4×4-meter grid (ie, the play area) was drawn on the floor using string and chalk, with grid lines drawn 1 meter apart. Participants were randomly assigned to start with either the high (n=18) or the low (n=18) body-involvement condition; the conditions were counterbalanced for the total individuals through an established randomization scheme obtained from the Research Randomizer website [87] run by one of the researchers involved in the study before the start of the experiments. After completing each gameplay mode, participants again answered the following self-reported questionnaires: the VAS-HP, VAS-SP, VAS-FE, VAS-SD, VAS-A, and STAI-Y1. The complete experience lasted about 40 minutes long (see Figure 1).

### Strategy of Data Analysis

Data were analyzed by means of a set of multivariate statistical tests. First, common assumptions (ie, normality, homogeneity of variance, and homoscedasticity) for multivariate analysis were assessed and procedures of data cleaning (ie, missing-values analysis and detection of uni- and multivariate outliers) were conducted. In general, no major violations to assumptions were found and a multivariate outlier was skipped. Multivariate outliers were identified by computing Mahalanobis distances, and the *P* value was set to be equal to .001.

In order to explore our research hypotheses, different series of generalized linear models (GLMs) were performed. For hypothesis 1, a GLM model for repeated-measures multivariate analysis of variance (MANOVA) was tested by including the four VAS measures related to positive (eg, happiness and surprise) and negative (eg, fear and sadness) emotions. Then, a second model was tested by including the two measures (eg, VAS-A and STAI-Y1 scores) of state anxiety. Such analysis is useful in measuring the effect of a *treatment* at different time points and in different groups. Furthermore, the GLM enabled evaluation of the main effect within and between the subjects as well as interaction effects between factors. Finally, the GLM estimated the magnitude of effect sizes for all variables. In this study, the GLMs were conducted in such a way that pre- and postexperiment measures were the within-subjects factors (ie, three levels), whereas the condition (ie, high vs low body-involvement video game) was the between-subjects factor. The interactive effect *within × between* was included in the model. For testing hypothesis 2, two GLMs were conducted in order to compare whether pre- and postexperiment differences in target variables (ie, positive emotion as well as negative emotion and anxiety) were statistically significant when low body-involvement and high body-involvement games were compared, once controlled for the order of presentation. For all tested models, effect size was reported (η^2^). According to Cohen [88], effect size should be considered small for values 0.20 and lower, medium for values between 0.21 and 0.50, and large for values of 0.51 and higher.

## Results

### Descriptive Characteristics of Participants

Gaming habits and the previous virtual reality experience of the sample are summarized in Table 1. Zero-order correlations between positive emotions, negative emotions, and anxiety scores are presented in Multimedia Appendix 2.

**Table 1:** Descriptive characteristics of the sample’s gaming habits and previous experience with virtual reality.

Characteristic	Mean (SD)
Hours spent gaming per week	9.5 (10.9)
Previous experience with virtual reality^a^	5.1 (2.8)

^a^Rated on a 7-point Likert scale ranging from 1 (not at all) to 7 (very much).

### Psychological Assessment

Starting from hypothesis 1 of this study, the GLM for the repeated-measures MANOVA was used to test whether video games played in virtual reality increased self-reported positive emotions (ie, VAS-SP and VAS-HP) and reduced negative emotions (ie, VAS-FE and VAS-SD) and perceived state anxiety (ie, VAS-A and STAI-Y1).

Generally speaking, the GLM testing differences among emotional scores (ie, VAS-SP, VAS-HP, VAS-FE, and VAS-SD) revealed a within-subjects statistically significant effect (*F*_8,27_=9.64, *P*<.001, η^2^=.741). On the contrary, the effect of the between-subjects condition (ie, low vs high body-involvement game as first stimulus) reported a nonstatistically significant effect (*F*_8,27_=0.56, *P*=.80, η^2^=.143), meaning that regardless of the order of presentation, there were differences in emotional levels between the baseline values and the scores obtained after a playing session. In particular, the domain of positive emotions reported statistically significant effects for both the VAS-SP (*F*_8,27_=7.93, *P*=.003, η^2^=.189) and the VAS-HP (*F*_8,27_=11.62, *P*<.001, η^2^=.255). In both cases, the scores on the high body-involvement game were higher if compared to both baseline and the low body-involvement game. Univariate tests in relation to negative emotions reported a statistically significant effect for both the VAS-FE (*F*_8,27_=5.13, *P*=.01, η^2^=.131) and the VAS-SD (*F*_8,27_=10.06, *P*<.001, η^2^=.228), meaning that negative-emotion levels were lower in high body-involvement games than what was reported at baseline and in low body-involvement games (see Multimedia Appendix 3).

**Figure 2:**
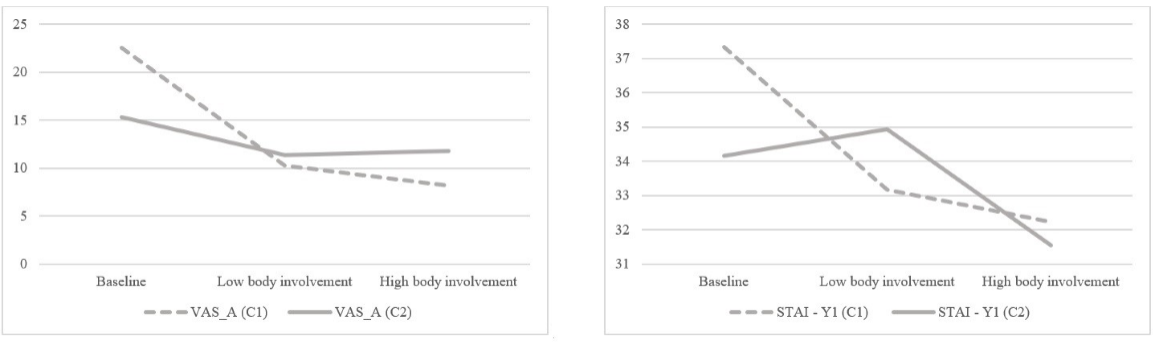
Mean scores of the Visual Analogue Scale-anxiety (VAS-A) and the State-Trait Anxiety Inventory, Form-Y1 (STAI-Y1). The VAS-A is measured on a scale consisting of a horizontal line, 10 cm in length, with scores ranging from 0 to 100. The STAI-Y1 is measured on a 4-point Likert scale ranging from 1 (not at all) to 4 (very much). C1: Condition 1, *Fruit Ninja VR* first; and C2: Condition 2, *Audioshield* first.

Regarding self-reported state anxiety (ie, STAI-Y1 and VAS-A), the results of the GLM revealed a within-subjects statistically significant effect (*F*_4,31_=4.58, *P*=.005, η^2^=.372). A nonstatistically significant effect was found in terms of presentation order between the different games (*F*_2,33_=0.01, *P*=.99, η^2^=.001). Univariate tests revealed that both the VAS-A (*F*_4,31_=8.01, *P*=.002, η^2^=.191) and the STAI-Y1 (*F*_4,31_=6.45, *P*=.003, η^2^=.160) reported statistically significant effects, meaning that the two measures of state anxiety converged in cross-validating the idea of a reduction of anxiety levels in players during virtual reality gaming sessions (see Figure 2). As a result, the findings supported the acceptance of hypothesis 1.

With regard to hypothesis 2, the GLM MANOVA was used to test whether, and to what extent, the psychometric scores of self-reported positive and negative emotions (ie, VAS-SP, VAS-HP, VAS-FE, and VAS-SD) and state anxiety (ie, VAS-A and STAI-Y1) were related to playing low body-involvement or high body-involvement games (see Table 2).

**Table 2:** Self-reported emotions and anxiety scores among subjects in the experimental conditions.

Questionnaire and conditions		Mean (SD)
**Visual Analogue Scale-happiness**		
	Baseline	62.3 (20.1)
	High body involvement	77.6 (20.4)
	Low body involvement	68.2 (24.2)
**Visual Analogue Scale-surprise**		
	Baseline	50.0 (31.6)
	High body involvement	66.9 (29.2)
	Low body involvement	57.8 (30.8)
**Visual Analogue Scale-fear**		
	Baseline	8.05 (11.7)
	High body involvement	1.72 (4.29)
	Low body involvement	2.69 (6.11)
**Visual Analogue Scale-sadness**		
	Baseline	10.1 (15.2)
	High body involvement	2.36 (5.91)
	Low body involvement	5.27 (10.6)
**Visual Analogue Scale-anxiety**		
	Baseline	18.8 (16.8)
	High body involvement	10.4 (13.6)
	Low body involvement	10.8 (12.6)
**State-Trait Anxiety Inventory, Form-Y1**		
	Baseline	35.7 (8.00)
	High body involvement	31.8 (7.44)
	Low body involvement	34.1 (7.91)

The GLM testing between-game differences in emotional scores revealed a within-subjects statistically significant effect (*F*_4,31_=3.86, *P*=.01, η^2^=.333). On the contrary, the effect of the between-subjects condition (eg, low body-involvement vs high body-involvement game as first stimulus) reported a nonstatistically significant effect (*F*_4,31_=0.41, *P*=.72, η^2^=.050), meaning that regardless of the order of presentation, there were differences in emotional levels between the two games. Univariate tests regarding positive emotions reported statistically significant effects for both the VAS-HP (*F*_4,31_=12.43, *P*<.001, η^2^=.268) and the VAS-SP (*F*_4,31_=6.83, *P*=.01, η^2^=.167). In both cases, the differential scores on the high body-involvement game were higher if compared to scores obtained in the low body-involvement game. Univariate tests in relation to negative emotions reported a statistically significant effect for both the VAS-FE (*F*_4,31_=3.47, *P*=.05, η^2^=.099) and the VAS-SD (*F*_4,31_=5.91 *P*=.05, η^2^=.148), meaning that decrements in negative-emotion levels were more relevant in the high body-involvement game than what was reported after the low body-involvement game.

Regarding state anxiety scores, the result of the GLM revealed a within-subjects, statistically significant effect (*F*_2,33_=48.25, *P*=.01, η^2^=.334). A nonstatistically significant effect was found in terms of presentation order between different games (*F*_2,33_=2.62, *P*=.09, η^2^=.132). Univariate tests revealed that especially for STAI-Y1 scores (*F*_2,33_=5.41, *P*=.05, η^2^=.137), playing in high body-involvement games lead to a greater reduction of anxiety levels. On the contrary, the VAS-A (*F*_2,33_=0.055, *P*=.82, η^2^=.002) did not report statistically significant effects. The results supported the acceptance of hypothesis 2, meaning that a greater increase in positive emotions, as well as a reduction of negative emotions and anxiety levels, were found in players after a high body-involvement virtual reality game session.

## Discussion

### Principal Findings

To summarize, the two main principal findings of this study are as follows:

1. Virtual reality video games appear to be effective tools to elicit positive emotions and to decrease negative emotions and state anxiety in individuals.
2. The level of body involvement of the virtual video game has an important effect in determining the ability of a game to improve positive emotions and decrease negative emotions and state anxiety of the players.

The results that emerged from this study appear to support the first main hypothesis (ie, the video games played in virtual reality will increase self-reported positive emotions and will be able to reduce negative emotions and perceived state anxiety). In particular, results of the GLM repeated-measures MANOVA revealed a statistically significant increase in the intensity of happiness and surprise and, in parallel, a significant decrease of fear and sadness reported by the users. Regarding the ability to improve anxiety levels in the players, the results showed a significant decrease in perceived state anxiety after game play, assessed with both the STAI-Y1 and the VAS-A.

Regarding the second main hypothesis (ie, the higher the body movement, the more the game will elicit stronger positive emotions and a more intense decrease in negative emotions and state anxiety), the results of the GLM MANOVA showed a greater efficacy by the high body-involvement game (ie, *Audioshield*) versus the low body-involvement game (ie, *Fruit Ninja VR*) to both elicit positive emotions and reduce negative emotions and state anxiety, as measured by the STAI-Y1.

### Potential for Virtual Reality Gaming to Elicit Positive Emotions and Decrease Anxiety

The ability of virtual reality content to elicit positive emotions and a state of relaxation has been widely demonstrated in relation to virtual environments designed ad hoc by researchers for emotional induction, both in healthy individuals (eg, [89-91]) and in patients suffering from different mental conditions, including anxiety disorders (eg, [92, 93]). This feature has been adopted extensively to improve individuals’ well-being, since, as stated by the broaden-and-build model [34], positive emotions provide the organism with nonspecific action tendencies that can lead to adaptive behavior, such as being more likely to interact with others or engage in creative challenges [94,95]. Therefore, as underlined by the positive technology approach [96], technology, including virtual reality, can be an effective tool to improve the quality of people’s personal experiences [97].

Interestingly, what emerges from this study shows that not only virtual content specifically created for emotional induction but also commercial content such as virtual reality video games can be effective in inducing positive emotions and decreasing negative emotions and anxiety in individuals. Such a result appears in line with what has been reported recently, with a few exceptions [24], about the ability of virtual reality video games to elicit positive emotions such as joy in the players and to do so with a more intense effect than that of desktop video games [2,6,21]. Besides, according to the results of this study, virtual reality video games are able to not only elicit positive emotions but also decrease negative emotions (ie, sadness and fear) and state anxiety in the players.

If the results of this study are confirmed by future research, they might represent positive evidence for the adoption of virtual reality games, not just for entertainment purposes but also in the mental health panorama. Given that positive emotions are linked strongly to the use of technological products and the user’s overall level of satisfaction with them—as underlined by *emotional design* [98,99], a conceptual framework that is largely adopted in the context of developing interactive technologies [100, 101]—virtual reality video games could be particularly appealing to users. Thanks to their being cost-effective, noninvasive, and nonassociated with stigma or with known side effects, virtual reality video games could offer many advantages compared to classic interventions for improving psychological well-being and decreasing anxiety, such as medication treatment, guided meditation, or cognitive behavioral therapy. In addition, virtual reality games also have several advantages compared to video games played on desktop displays, including the characteristic of requiring higher involvement both at the motor and cognitive levels and a greater engagement compared to nonimmersive games [17,55]. They also offer the possibility of collecting a wide variety of data, such as those about the player’s movement within the virtual environment.

Furthermore, the fact that, in this study, these results were observed after a short period of play (ie, about 5 minutes for each video game) could suggest that even very brief interventions with games in virtual reality can be effective ways to increase players’ positive emotions and decrease negative emotions and state anxiety. Since the length of the interventions with video games to enhance positive emotions and relaxation vary in the literature [56], with an average of 20-25 minutes of total gameplay (eg, [46,47]), the results obtained by this study could represent an element to broaden the reflection on this important topic, and further research is needed.

### Significance of the Body’s Involvement on Players’ Psychological Benefits

Differences were observed in this study regarding the ability to elicit positive emotions and decrease negative emotions and state anxiety, as measured by the STAI-Y1, depending on the level of body movement requested by the game; this offers interesting insights on the role of body involvement in determining the psychological benefits of video games.

In particular, we observed a greater efficacy of the game that required high body involvement (ie, *Audioshield*) compared to the game with low body involvement (ie, *Fruit Ninja VR*). Such results appear to be consistent with previous literature reporting that an increased involvement of the body can afford the player not only a greater level of enjoyment but also a stronger emotional experience [69,70]. In the specific case of virtual reality video games, it could be hypothesized that this effect is related to the ability of body movements to enhance sense of presence [102]. However, other studies have not reported a direct effect of body involvement on sense of presence during virtual reality content [103]. Therefore, future studies should investigate this topic further.

If the results observed in this study are confirmed by future studies, it could represent valid evidence that virtual reality video games, especially those that require a high level of involvement of the player's body, promote emotional well-being in individuals. For example, it would be interesting to test the effects of a virtual reality-based exergame, a type of video game that has proven to be effective for emotional relief and for its antidepressant effect when played on desktop displays [65-67,104, 105].

### Limitations

Although the results of this study could be interesting for their possible applications, this research has some important limitations that prevent a robust generalization of the findings. First, the design of the study did not include a control group (ie, a no-video-game condition). Second, in this study no physiological measure related to emotional responses (eg, heart rate variability and skin conductance responses) and/or state anxiety (eg, parasympathetic activity and cortisol level) were examined. Third, the video games were played for short time periods (ie, about 5 minutes each), and only short-term changes were considered. Finally, it is important to underline the small sample size and the specificity of the included sample (ie, young adults who played several hour per week and had medium-level knowledge of virtual reality systems). All in all, even if the results of this research can be considered promising, due to major limitations, including the probability of being underpowered, robust conclusions were still hard to achieve. In future research, it would be interesting, for example, to widen the sample in order to balance participants on the basis of their level of experience with video games and to collect baseline measures on whether they enjoy exercise in general.

### Conclusions

On the basis of what emerged in this study, and as underlined in previous research (eg, [56,106]), the research question no longer seems to need to ask whether video games are good or bad for the mental health of the individual, but rather seeks to investigate how their specific characteristics (eg, genre, display device, and duration and frequency of playing) may have an impact on the efficacy of the game to improve players’ well-being. In the future, it would be rather interesting to deeply investigate this topic; it would also be interesting to consider the specific characteristics of the players (eg, gender and gaming habits). In addition, future research should investigate the adoption of other commercial virtual reality systems with different characteristics in terms of immersion and interactions with the game, including other off-the-shelf virtual reality systems, such as Oculus Go (Facebook Technologies) or PlayStation VR (Sony Interactive Entertainment). These products are very appealing since, unlike the system tested in this study, they could be used easily even by nonexpert operators and are more budget friendly.

## Appendix

Multimedia Appendix 1 Experimental setting of the study.

Multimedia Appendix 2 Zero-order correlations between positive emotions, negative emotions, and anxiety scores (N=36).

Multimedia Appendix 3 Mean scores of the positive and negative emotional states. Positive emotions are reported in the left-hand column and negative emotions are reported in the right-hand column. C1: Condition 1, Fruit Ninja VR first; and C2: Condition 2, Audioshield first.

## Abbreviations

EEG: electroencephalogram
FPS: first-person shooter
GLM: generalized linear model
HD: high definition
HDMI: high-definition multimedia interface
HMD: head-mounted display
MANOVA: multivariate analysis of variance
MSI: Micro-Star International
PET: positron emission tomography
STAI-Y1: State-Trait Anxiety Inventory, Form Y1
TMT: Trial Making Test
VAS: Visual Analogue Scale
VAS-A: Visual Analogue Scale-anxiety
VAS-FE: Visual Analogue Scale-fear
VAS-HP: Visual Analogue Scale-happiness
VAS-SD: Visual Analogue Scale-sadness
VAS-SP: Visual Analogue Scale-surprise
