# Supplementary figures and images for: Virtual Reality Games and the Role of Body Involvement in Enhancing Positive Emotions and Decreasing Anxiety: Within-Subjects Pilot Study
Source: JMIR Serious Games. 2020 Jun 17;8(2):e15635. doi: 10.2196/15635 (PMC7330737; doi:10.2196/15635)

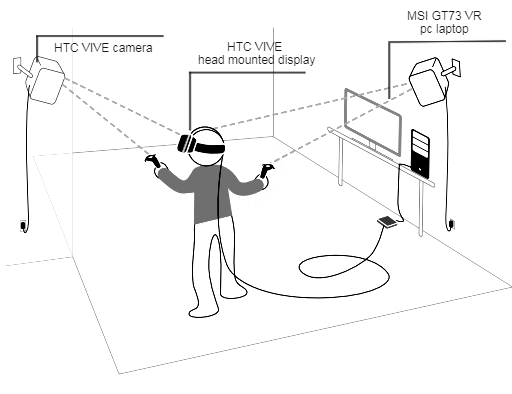

Supplement: Multimedia Appendix 1 [file games_v8i2e15635_app1.png]

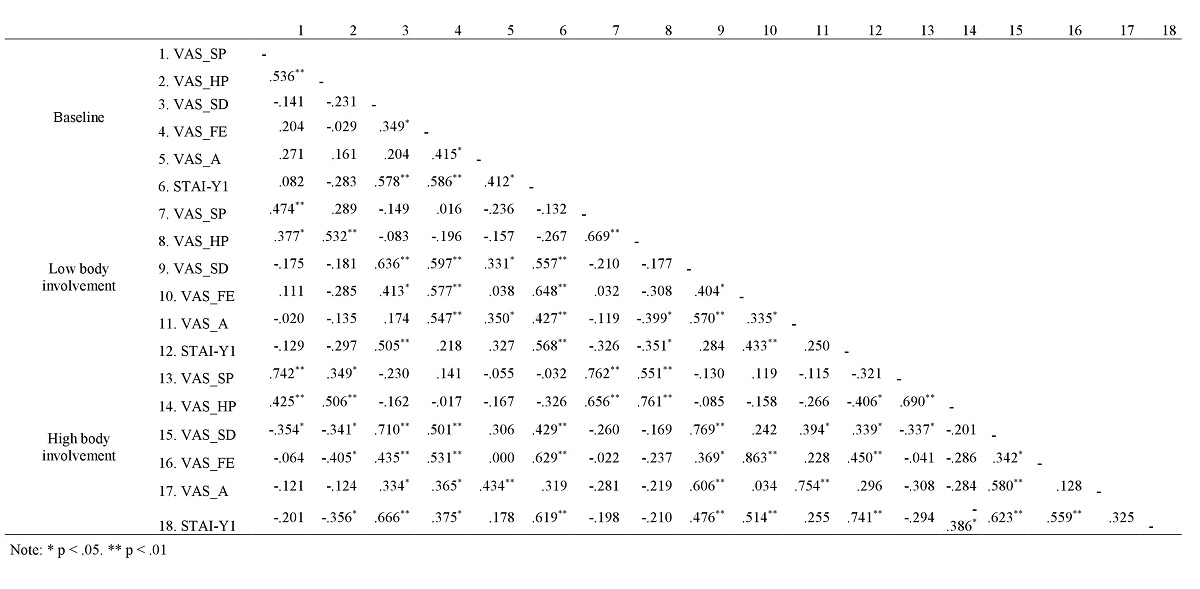

Supplement: Multimedia Appendix 2 [file games_v8i2e15635_app2.png]

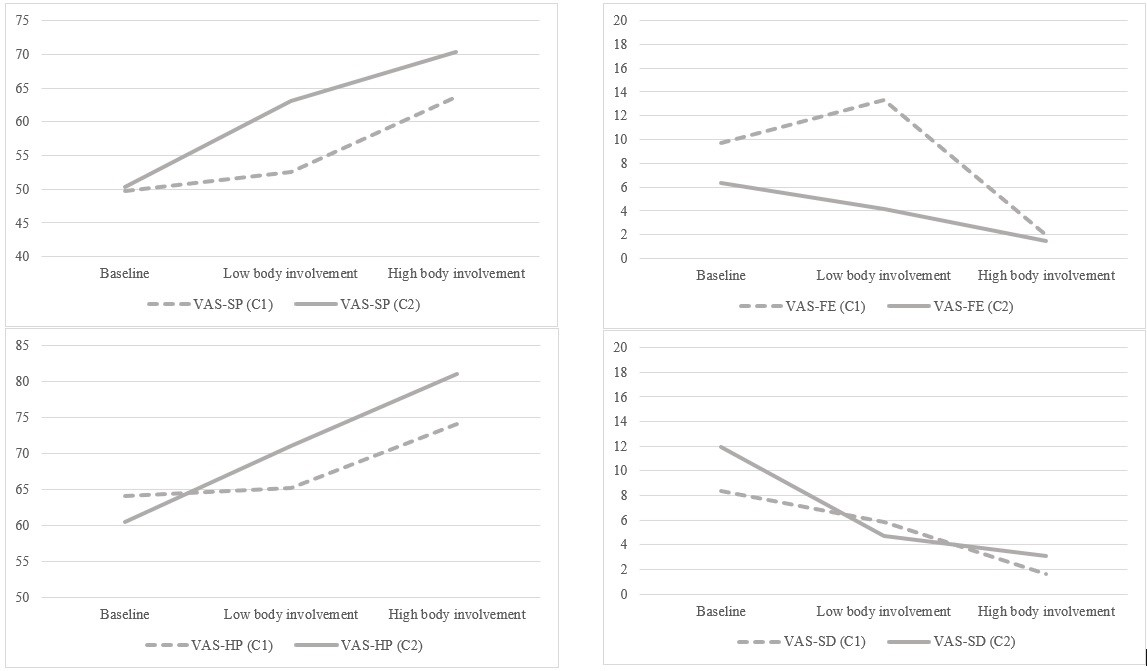

Supplement: Multimedia Appendix 3 [file games_v8i2e15635_app3.png]
